# Tetracycline-mediated fluorescence correlates with passage number and β-galactosidase activity in HeLa and HEK293T cells

**DOI:** 10.1242/bio.059691

**Published:** 2023-06-28

**Authors:** Chileab Redwood-Sawyerr, Darren N. Nesbeth

**Affiliations:** Department of Biochemical Engineering, University College London, Bernard Katz Building, London WC1E 6BT, UK

**Keywords:** Senescence, Tetracycline, Marker, Cellular, HeLa, HEK293T

## Abstract

We report data consistent with tetracycline-mediated fluorescence having the potential to be an effective marker of senescence in immortalised cells. HeLa cells that had previously undergone more than 20 passages were transiently transfected with a plasmid encoding a novel tetracycline-inducible transgene featuring an open reading frame for green fluorescent protein. While characterising the performance of this plasmid and transfection procedure, HeLa cell fluorescence was observed to result from incubating cells with media containing 2 μg/ml tetracycline alone, without plasmid or transfection reagent. To investigate this phenomenon further, HeLa and HEK293T cells were purchased from a tissue culture collection and after cultivation over 4-23 passages, incubated with media containing 2 μg/ml tetracycline. For both cell lines, tetracycline-mediated fluorescence increase correlated with passage number increase. This effect in HeLa and HEK293T cells was also borne out by expression of β-galactosidase activity, an imperfect but widely used marker of cellular senescence. These data suggest tetracycline may have utility as a marker of cellular senescence in immortal cells and can inform future investigation and validation of this previously unreported application for this reagent.

## Teaser

**Summary:** This paper reports data consistent with tetracycline-mediated fluorescence having the potential to be an effective marker of senescence in immortalised cells.

## INTRODUCTION

Tetracycline (Chemical Abstracts Service number 60-54-8) is a fluorescently active molecule used widely as an antibiotic (Chopra and Roberts, 2001), a clinically-approved fluorochrome reagent for labelling human bone osteocytes *in vivo* and as a label for identifying pathologically calcified tissues in histological samples (Pautke et al., 2010). To date no applications have been reported for tetracycline in the field of cellular senescence.

The genetic toolbox for engineering mammalian cells includes transgenes incorporating tetracycline-inducible promoters, referred to as ‘Tet-On’ systems (Wieland and Fussenegger, 2012). We initially sought to characterise a novel, plasmid-encoded, ‘Tet-On’ promoter controlling transcription of an open reading frame for green fluorescent protein (GFP). We tested the plasmid via transient transfection of the HeLa cell line, an adherent, immortal, tumour-derived line used commonly in clinical research and biotechnology applications (Kast et al., 2021).

## RESULTS

### Tetracycline-associated cell fluorescence

**Fig. 1.**
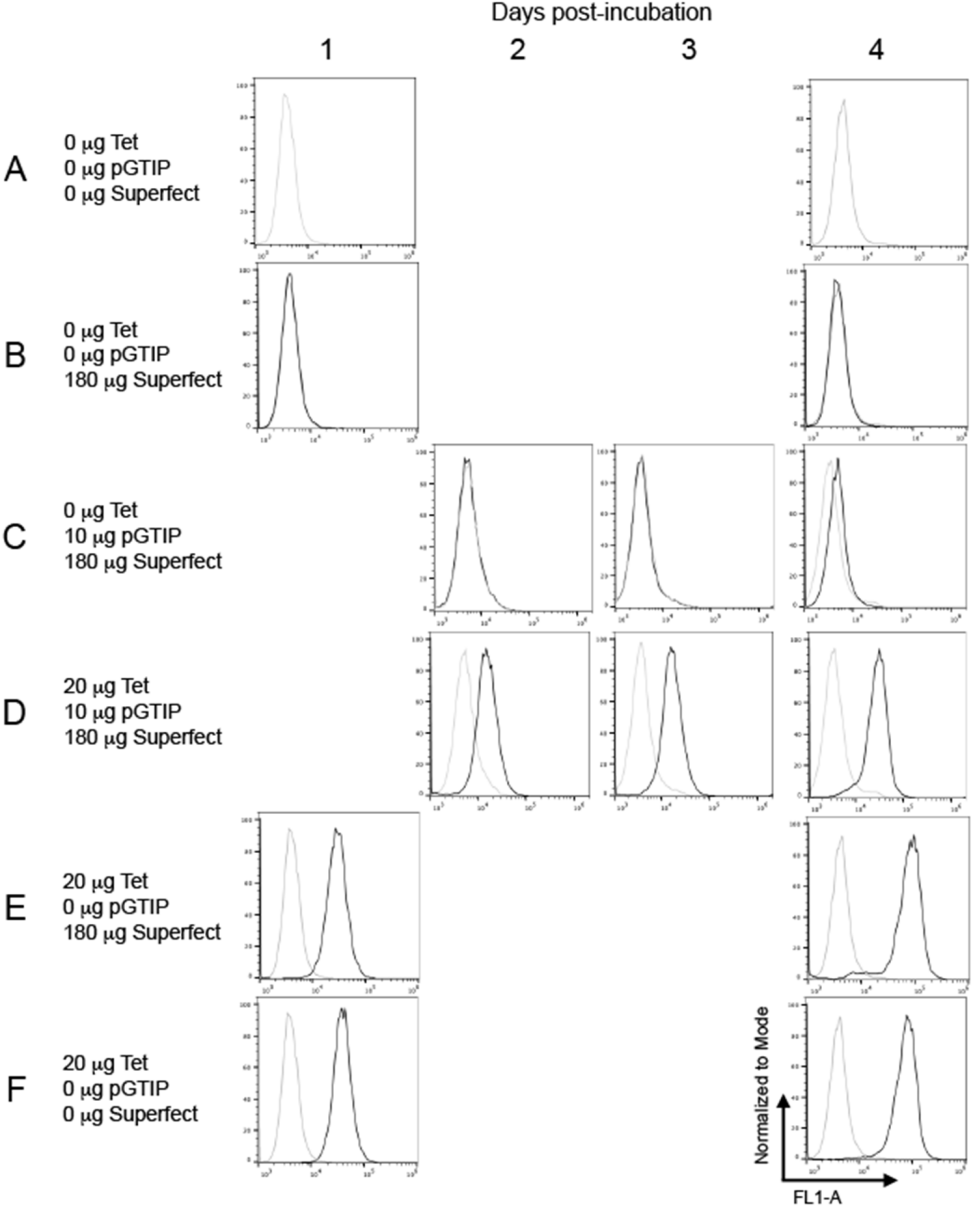
**Cell fluorescence observed after treating HeLa cells with tetracycline alone.** HeLa cells that had previously undergone >20 cell culture passages were grown to 80% confluence in 10 ml 10% v/v FBS DMEM in 10 cm tissue culture Petri dishes and on Day 0 (no data panels) either left untreated (row A) or treated by addition of the indicated mass of the plasmid pGTIP and/or Superfect (rows B-E) for 3 h. Columns 1-4 at the top of the data panel indicate how many days after this 3-h incubation flow cytometric data was captured. After the 3-h incubation period, for all dishes, media was removed and replaced with either 10 ml unsupplemented media (rows A-C) or 10 ml media containing 20 μg tetracycline (rows D-F) for 24 h. After this 24-h period, all media was removed and replaced with 10 ml unsupplemented media followed by further incubation for 2, 3 or 4 days (columns 2, 3 and 4) or immediate trypsinisation for flow cytometry (column 1). In the flow cytometry data panels, grey line data was from untreated cells, black line data from cells treated as indicated in the row title. All cell-count data (Y axis) was normalised to mode values and fluorescence data (X axis) was via the FL1-A laser (515-545 nm). Data in the 2-day and 3-day columns are each from a single experiment and data in the 1-day and 4-day columns are each representative of *n*=4 independent repeat experiments.

Fig. 1 shows results from our initial attempt to transiently transfect HeLa cells with a plasmid incorporating a ‘Tet-on’ system for controlling GFP expression. Rows A-C of Fig. 1 show results from untreated cells, cells incubated with Superfect only, and cells incubated with Superfect and plasmid only, showing no marked increase in fluorescence. When a 24-h incubation with media supplemented with 2 μg/ml tetracycline was introduced, post-transfection, a marked increase in fluorescence was observed (Fig. 1, row D). In isolation, rows C and D of Fig. 1 would appear to evidence the expected ‘Tet-On’ phenotype, with the marginal increase in fluorescence on Day 4 in Row D due to leakiness of the Tet-On promoter system. However, control experiments showed that omitting plasmid (Fig. 1, row E) and omitting both plasmid and Superfect (Fig. 1, row F) still resulted in marked fluorescence when cells were incubated with 2 μg/ml tetracycline. To determine the repeatability of the observed fluorescence in the absence of plasmid, three further independent repeats of the experiments in the 1-day and 4-day columns (Fig. 1) were performed. All repeats showed the same pattern of fluorescence appearance in the absence of plasmid and transfection reagent and the presence of tetracycline.

The fluorescence observed in Fig. 1, Rows E and F, prevented confirmation of a plasmid-encoded ‘Tet-On’ function as they suggested all observed fluorescence, including Fig. 1, Row D, resulted from incubation with 2 μg/ml tetracycline alone. We considered that the observations in Fig. 1 may suggest a previously unreported property of senescent immortal cells; namely that of increased association with tetracycline. To test this hypothesis, we purchased new batches of the immortal cell lines HeLa and HEK293T, from the American Type Culture collection (ATCC).

### Tetracycline-associated cell fluorescence as a function of passage number

**Fig. 2.**
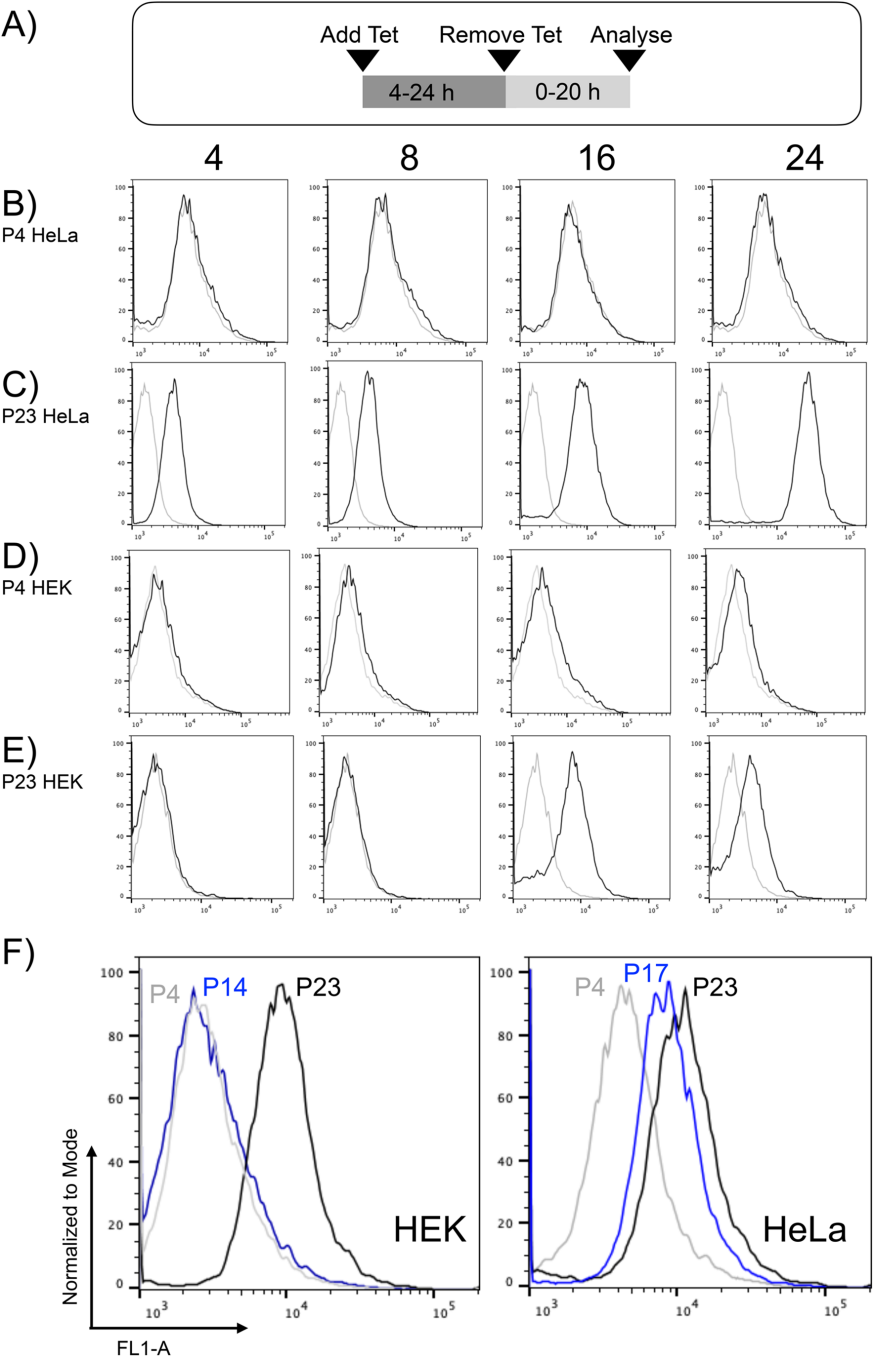
**Beta-galactosidase activity and tetracycline-mediated fluorescence both correlate with HEK and HeLa cell passage number.** HeLa cells that had undergone 4 (P4) or 23 (P23) passages were incubated in 10% v/v FBS DMEM (rows B and C respectively) either in the absence (grey data profiles) or presence (black data profiles) of 2 μg/ml tetracycline in 100 mm tissue culture dishes for 4, 8, 16 or 24 h, as indicted by column labels. In the case of 24-h incubation with tetracycline-containing media, the media was not changed prior to flow cytometry. For 4, 8 and 16-h tetracycline incubations, media was replaced with tetracycline-free media for 20, 16 and 8 h respectively (illustrated in panel A), to ensure a total of 24 h had elapsed since initial addition of tetracycline-containing media. P4 and P23 HEK cells (rows D and E respectively) were treated in the same manner as the HeLa cells. In the flow cytometry data panels, grey line data was from untreated cells, black line data from cells incubated with 2 μg/ml tetracycline for the time in hours indicated in the column title. In panel F, populations of HEK293T (left hand plot) and HeLa (right hand plot) cells, of the indicated passage number, were cultivated in 100 mm dishes then fixed with paraformaldehyde prior to incubation with the ‘Green Probe’ fluorigenic substrate. Cells were analysed by flow cytometry where grey line data was from P4 cells, blue line data from P14 HEK cells or P17 HeLa cells and black line data was from P23 cells. All cytometric data was plotted as in Fig. 1. In rows B-to-E, data in the 8-h and 16-h columns are each from a single experiment and data in the 4-h and 24-h columns are representative of *n*=4 independent experiments. In row F, all data are representative of *n*=4 independent repeat experiments.

We seeded ATCC-purchased HeLa and HEK cell lines the day before to give confluence of approximately 75% on the day of experimentation. Media was then replaced with fresh media either with or without 2 μg/ml tetracycline. Tetracycline-containing media was then left on the cells for up to 24 h before being removed prior to flow cytometry (Fig. 2A). This procedure (Fig. 2B-2E) was performed for cells that had undergone 4 (P4) or 23 passages (P23) since cryorevival.

P23 HeLa cells exhibited markedly greater fluorescence than P4 HeLa cells after 16 and 24 h in the presence of tetracycline (Fig. 2B,C). P23 HeLa cells also showed a clear difference in fluorescence between cells that had been incubated with unsupplemented media and those incubated with media containing 2 μg/ml tetracycline (Fig. 2C, compare grey and black data lines). This difference was greatest after 24 h incubation with tetracycline. No such difference was observed with the P4 Hela cells (Fig. 2B, compare grey and black data lines).

P23 HEK cells also showed greater fluorescence than P4 HEK cells after 16 and 24 h in the presence of tetracycline (Fig. 2D,E). P23 HEK cells showed the clearest difference in fluorescence between cells that had been incubated with and without tetracycline, with the greatest difference observed after 16 h incubation (Fig. 2E, compare grey and black data lines). Three further independent repeats of the experiments in the 4-h and 24-h columns (rows B-E) confirmed the replicability of the observed greater fluorescence with greater passage number.

### Beta galactosidase activity as function of passage number

Observations in Fig. 2B-E were consistent with tetracycline-mediated fluorescence increasing as passage number increased. This may be due senescence-mediated changes to the cell surface that increase net affinity for tetracycline, given that cellular senescence can remodel the surface of cells (Kim et al., 2017; Madsen et al., 2017).

We tested whether HeLa or HEK passaging correlates with β-galactosidase activity, a widely used marker of cellular senescence (Lee et al., 2006). We used the CellEvent Senescence Green Flow Cytometry Assay Kit from Thermo Fisher Scientific to measure β-galactosidase in HEK cells that had undergone 4 (P4), 14 (P14) or 23 passages (P23) since cryorevival and equivalent HeLa cells at P4, P17 and P23 (Fig. 2F). The kit features the ‘Green Probe’ substrate which fluoresces upon cleavage by β-galactosidase.

HeLa cell passage number showed a linear relationship with β-galactosidase activity (Fig. 2F, right hand data panel) with β-galactosidase activity increasing after 17 and again after 23 passages, compared to the level at 4 passages. For HEK cells β-galactosidase activity markedly increased after 23 passages but there was effectively zero increase from passage 4 to 14 (Fig. 2F, left hand data panel). These observations were consistent across *n*=4 independent experimental repeats.

## DISCUSSION

We understand this observed property of tetracycline with respect to the senescence of immortalised cell lines to be novel. We anticipate tetracycline could be exploited to rapidly assess senescence of HeLa or HEK-based cell lines of uncertain provenance and passage number. Further characterisation and validation will be needed to establish whether senescence-based tetracycline-binding is broadly applicable to other biological settings.

## MATERIALS AND METHODS

We initially used HeLa cells from a historic cell bank, cultivated using standard techniques with 10% v/v foetal bovine serum in High-Glucose/GlutaMAX Dulbecco's Modified Eagle's Medium (Life Technologies, Thermo Fisher Scientific, Waltham, MA, USA) in capped-vented T175 flasks (Corning Limited, Union City, CA, USA). Cells were plated in a media solution of typically 2×10^5^ cell/ml in static incubators at 37°C, 5% CO_2_, and passaged upon reaching 80% confluence.

This initial HeLa cell line was estimated to have undergone more than 20 consecutive passages. Cells were seeded at 5×10^5^/ml and yielded a confluency of approximately 75% after overnight growth. Media was changed to serum-free Ultraculture media (Lonza, UK), and plasmid DNA plus Superfect (Qiagen, MD, USA) added to cells. Cells were prepared for analysis by flow cytometry 1, 2, 3 or 4 days after transfection. For cytometry cells were washed with 10 ml phosphate-buffered saline (PBS), incubated with 2 ml trypsin-EDTA (Merck-Millipore, Germany) at 37°C for 5 min then 2 ml PBS added addition for resuspension. Cells were then centrifuged and re-suspended in 1-2 ml PBS to remove residual trypsin. Fluorescence analysis was performed using the BD Accuri C6 Plus (BD Biosciences) device, counting 20,000 ungated events. Histogram data analysis was performed using absolute mean fluorescence intensity (MFI) with the FL1-A laser, which can detect both the emission peaks of tetracycline (529 nm) and GFP (509 nm).
